# Supplementary material for: Early lyophilized cryoprecipitate enhances the ADAMTS13/VWF ratio to reduce systemic endotheliopathy and lessen lung injury in a mouse multiple-trauma hemorrhage model
Source: J Trauma Acute Care Surg. 2023 May 22;95(2):S137–43. doi: 10.1097/TA.0000000000004065 (PMC10389395; doi:10.1097/TA.0000000000004065)
Supplement: Supplementary file 1 [file jt-95-s137-s001.docx]

**INTRODUCTION**

Hemorrhagic shock (HS) remains the leading cause of early deaths among severely injured patients in both civilian and military settings.^1^ Through the adoption of hemostatic resuscitation- based strategies, mortality has decreased.^2^ However, such strategies may not be available in the battlefield, at the point of injury, or during prolonged transport where they would be most advantageous. As future areas of military operation are anticipated to require prolonged periods of field care, delays in access to blood products could place injured soldiers with severe injuries and shock at increased risk for early death and for survivors increased risk of organ dysfunction. Similarly, access to blood products in the prehospital setting or in rural areas remains limited in the civilian setting.
 Hemorrhagic shock has clearly been associated with the endotheliopathy of trauma (EoT)^3^ which occurs rapidly following trauma^4^ and is defined by endothelial cell, coagulation, and immune dysfunctions. Reversing or mitigating the EOT is believed to a contributing factor in the improved outcomes in hemorrhagic shock patients receiving early plasma transfusion, providing benefit beyond that of improving hemostasis .^5^ As additional data is acquired related to the mechanisms by which hemostatic resuscitation repairs the endothelium, there is an opportunity to expand studies beyond those of just plasma and to now include plasma alternatives such as cryoprecipitate..

Cryoprecipitate processed from fresh frozen plasma is enriched with fibrinogen, von Willebrand factor (VWF), factor VIII, factor XIII and fibronectin, as well as ADAMTS13 (a disintegrin and metalloprotease with thrombospondin type motifs-13), thus potentially providing similar or augmented endothelial protection to fresh frozen plasma (FFP) alone. Fibrinogen is enriched in cryoprecipitate and has been identified as the essential protein in plasma responsible for its protective effects.^6–8^ Wu, et al has shown that fibrinogen protects the endothelium by stabilizing cell surface syndecan-1.^6^ They subsequently demonstrated that this binding activated a PAK1 mediated signaling pathway that decreased stress fiber formation of the endothelium and thus enhanced barrier protection.^7^ VWF is also a key constituent in cryoprecipitate as it plays a critical role in hemostasis and is enriched in cryoprecipitate compared to fresh frozen plasma (FFP).^9,10^

Following trauma and other inflammatory disease states, however, VWF is released from injured and activated endothelial cells and platelets through granule exocytosis in a pathologic hyperadhesive form.^11^ Recent clinical evidence suggests that this hyperadhesive form of VWF may contribute to endothelial cell dysfunction and organ injury.^12,13^ Additionally, ADAMTS13, the metalloprotease that cleaves VWF to reduce its hyperadhesive activity, is low in injured patients.^12,14-16^ We *hypothesized* that the early use of cryoprecipitate would be effective as an endothelial protector by both supplementing physiologic ADAMTS13 to reduce pathologic VWF and to reverse the EOT. Using an animal model, we evaluated a pathogen-reduced lyophilized cryoprecipitate (LPRC) that could expedite the early administration of cryoprecipitate to wounded warfighters in the battlefield.

**Methods**

### Donor plasma and cryoprecipitate

Plasma was obtained from healthy donors through the Bonfils/Vitalant Blood Bank Research Donor Program, Denver, Colorado. Per standard blood bank procedures, plasma was frozen and stored at −20°C within eight hours until ready for testing. Pathogen-reduced cryoprecipitated AHF (conventional pathogen reduced cryoprecipitate, CC), and lyophilized pathogen-reduced cryoprecipitate (LPRC) products were supplied by Cerus Corporation (Concord, CA) and similarly stored at -20°C within eight hours. Lyophilized cryoprecipitate was reconstituted according to the manufacturer recommendations at the time of experimentation and used immediately. We acknowledge that human product is being transfused into mice. However, Peng, et al detected no differences in lung indices when comparing mouse to human plasma in a mouse model of hemorrhagic shock.^17^

**Animals**

Wild-type male C57BL/6J mice (8-12 weeks of age) were obtained from Jackson Laboratories (Bar Harbor, Maine) and housed in pathogen-free conditions for one week prior to experiments. This study was approved by the Institutional Animal Care and Use Committee and the Animal Care and Use Review Office (ACURO) of the US Army and conformed to the ARRIVE guidelines. The ARRIVE checklist is included in the Supplemental Digital Content (<http://links.lww.com/TA/D54>).

**Mouse model of polytrauma and uncontrolled hemorrhage**

Mice underwent isoflurane anesthesia. The femoral artery and vein were cannulated for continuous blood pressure monitoring and blood withdrawal or fluid administration. A unilateral midshaft tibia fracture was induced by blunt force and the ipsilateral gastrocnemius muscle was crushed with a clamp for 20 minutes.^18^ Next, a midline laparotomy was made. Pre-weighed gauze pads were placed in the peritoneal cavity away from the liver. The liver was isolated and an injury created by sharply transecting 50-60% of the left lobe.^19^ The lacerated segment was removed and then weighed. The abdomen was quickly closed. Additional blood was removed/returned using the femoral catheter to maintain mean arterial pressure (MAP) at 35 +/- 5 for 60 minutes. The mice then were randomly resuscitated with either CC, LPRC, FFP, or Lactated Ringers (LR) to a MAP of 55±5 mm, which was maintained for three hours to simulate prolonged field care.^20^ Sham mice underwent anesthesia and vessel cannulation but no surgical procedures. Mice were then euthanized by cardiac puncture and blood, tissue and bronchoalveolar fluid harvested.

**Lung Permeability**

The trachea was cannulated and bronchoalveolar lavage (BAL) fluid was collected by three injections of 0.4 mL of phosphate-buffered saline into the left lung. BAL fluid was centrifuged at 10,000xg at 4°C for 10 minutes. The supernatant was stored at −80°C. Total protein in the BAL fluid was measured with the BCA Protein Assay (ThermoFisher Scientific, Waltham, MA) as a surrogate marker for vascular permeability.^21^

**Lung histopathologic injury**

Lung tissue embedded in optimal cutting temperature compound (OCT) at the time of sacrifice and stored at −80°C. Lung tissue was sectioned and stained with hematoxylin and eosin (H&E) and scored on a three-point scale for alveolar thickness, capillary congestion, and cellularity as described by Hart, *et al*. and others.^22,23^ The overall lung injury score was calculated in a blinded fashion by averaging the three parameters.

**Lung syndecan-1 immunostaining**

To detect cell surface syndecan-1, lung tissue were sectioned and stained with an anti-mouse syndecan-1 antibody (Santa Cruz Biotechnology, CA) and Alexa Fluor 488 goat anti-mouse IgG (Invitrogen, CA).^24^ Random images were taken from each section with a fluorescent microscope at 100x using Infinity2-1R camera. The images were quantified using Quantity one software (Bio-RAD). Results are reported as relative fluorescent units (RFUs).

**Systemic Parameters**

Blood was obtained via cardiac puncture at the time of euthanasia. Plasma was separated from whole blood and frozen at -80º until time of assays. VWF antigen (VWF-Ag), ADAMTS13 antigen (ADAMTS13-Ag), and circulating syndecan-1 were measured by ELISA according to manufacturer instructions (ThermoScientific and Boster Bio, USA).

**Liver mRNA**

To examine the potential contribution of endogenous expression of ADAMTS13 to our findings, the right lobe of the liver was collected at time of euthanasia and flash frozen and stored at -80º. The liver tissue, as the primary site for ADAMTS13 production,^25^ was tested for mRNA expression in the different groups. ADAMTS 13 was detected using primers (forward: CTTATCACCCTCTCTGACTC; reverse: GTCAAACCTGGTGATATAGAG). The results were normalized to sham values and expressed as fold change.

**Statistical analysis**

Data were analyzed by one-way analysis of variance (ANOVA) with Bonferroni correction for multiple comparisons; p-values <0.05 were considered statistically significant. MAP data were analyzed by ANOVA with Tukey post hoc with multiple comparisons corrections. Quantitative data were expressed as mean ± standard deviation (SD).

**RESULTS**

**Physiologic Parameters**

Overall mortality was 29.8% for the resuscitation groups. There were no significant differences in mortality between resuscitation groups, however the study was not powered to detect difference (**Table 1**). It is possible that cryoprecipitate infusion itself could have contributed to the lack of mortality benefit. However, a recent study be Zeineddin, et al examined organ dysfunction at 72 hours post-injury after using cryoprecipitate products in a mouse model of hemorrhagic shock and showed no adverse effects^26^. The extent of hemoperitoneum following liver injury and total blood removed to reach shock state also did not differ between groups (**Table 1**). Resuscitation volumes to achieve our hypotensive MAP target were similar between FFP, CC, and LPRC groups but significantly higher in the LR group. (**Table 1**). Mean arterial pressure was similar across resuscitation groups at baseline and the end of shock by design but by 180 minutes of hypotensive resuscitation, MAP of FFP, CC and LPRC groups were all higher than LR (**Figure 1).**

**Lung Dysfunction Lessened by Cryoprecipitate Products**

Mice that received LR had a higher lung histopathologic injury score than the other resuscitation groups (LR 2±0.2, FFP 1.45±0.2, CC 1.3±0.1, LPRC 1.4±0.1; p<0.01), which were comparable to sham mice (**Figures 2A**).

BAL protein was measured as an indicator of lung permeability, which was also significantly higher in LR (148±29 *µ*g/ml) compared to FFP (113±30 µg/ml) and CC (108±34 µg/ml) while BAL protein levels of mice receiving LPRC (85±31 µg/ml) were further reduced to the level similar to that of sham mice (64±34 µg/ml, p=0.34, **Figure 2B**).

We hypothesized that the improvement in pulmonary vascular permeability with cryoprecipitate products may be associated with protecting the expression of syndecan-1 on pulmonary endothelial cells, similar to what has been shown for FFP.^20^ Indeed, both CC (6505±1918 RFUs) and LPRC (8445±2210 RFUs) increased pulmonary syndecan-1 immunostaining to the levels similar to shock mice receiving FFP (12,332±1433 RFUs) and shams (9401±2499 RFUs) while LR-resuscitated mice (2244±1070 RFUs) had a significant decrease in endothelial expression of syndecan-1 (**Figure 3).**

**Cryoprecipitate Products Improve Indices of Endotheliopathy**

Shed syndecan-1 ectodomain (soluble syndecan-1) was measured as an indicator of systemic endotheliopathy. Soluble syndecan-1 was higher in the LR group (LR 4.8±0.9 ng/ml) compared to other groups (Sham 3.2±0.6, FFP 2.7±0.7, CC 3.4±1.2, and LPRC 3±1 ng/ml, p<0.01, **Figure 4**).

VWF-Ag showed a significant increase after LR resuscitation (3.1±0.77 ng/ml) compared to sham mice (1.9±0.3 ng/ml) and shock mice receiving FFP (1.2±0.59 ng/ml) or CC (1.76±0.55 ng/ml). VWF-Ag was further reduced in mice receiving LPRC (0.69±0.33 ng/ml). ADAMTS13-Ag levels were significantly lower in LR resuscitated mice (217±105 ng/ml) compared to sham mice (511±131 ng/ml), while shock mice receiving CC increased ADAMTS13 to 583±126 ng/ml, comparable to that of sham mice. There was no significant difference in ADAMTS13-Ag between the FFP (316±55 ng/ml) and LRPC (342±107 ng/ml) mice compared to LR. To determine if differences in ADAMTS13 synthesis in the liver contributed to these results, liver mRNA levels were assessed. There were no significant differences between groups (Sham 1, LR 1.36, FFP 0.84, CC 0.7, LPRC 1.1 fold change), suggesting that changes in circulating levels of ADAMTS13 were attributable to the resuscitative agents.

Lastly, to evaluate the relationship between circulating levels of ADAMTS13 and VWF, we calculated the ratio of these two parameters. The ratio of ADAMTS13-to-VWF was markedly reduced in LR resuscitated mice compared to sham mice, suggesting a kinetic ADAMTS13 deficiency. In contrast, this ratio was restored back to the baseline levels in shock mice receiving FFP or CC. LPRC further increased the ratio compared to all other groups (**Figure 5**).

**DISCUSSION**

In the current study, we demonstrated that uncontrolled hemorrhagic shock and polytrauma in our mouse model resulted in worsening indices of endotheliopathy, as defined by higher systemic levels of circulating syndecan-1 and VWF, as well as lower levels of ADAMTS13. Conventional and lyophilized pathogen-reduced cryoprecipitate improved systemic endotheliopathy, similar to FFP, with a decrease in syndecan-1 shedding and augmentation of the ADAMTS13:VWF ratio to a homeostatic level. The latter ensures that VWF maintains its hemostatic activity but is no longer prothrombotic. The finding that increasing VWF cleavage protected mice against trauma/shock-induced endotheliopathy further suggests that uncleaved VWF is not only a marker for endotheliopathy, but also causing it. Mice receiving LPRC had the highest ADAMTS13-to-VWF ratio, primarily driven by a reduced VWF level. The changes in circulating endothelial markers were associated with improvements in lung permeability, as shown by histopathological markers and syndecan-1 expression. The LPRC resuscitation resulted in the lowest lung permeability, suggesting additional protection afforded by this product.

Barry, et al have shown in-vitro that cryoprecipitate attenuates thrombin-induced hyperpermeability and breakdown of endothelial adherens junctions comparable to fresh frozen plasma and Wu, et al reported that pathogen-reduced cryoprecipitate had similar endothelial protective effects to FFP.^27,28^ Additionally, in an in-vivo model of controlled hemorrhage in mice, conventional cryoprecipitate and FFP decreased lung injury, inflammation and permeability.^27^ The current study expanded on these novel findings but used a polytrauma model of uncontrolled hemorrhage, muscle crush and tibia fracture and a MAP based resuscitation strategy rather than a fixed volume strategy and extended investigation into lyophilized cryoprecipitate. We again demonstrated that cryoprecipitate protected the endothelium as FFP does.

Conventional cryoprecipitate-antihemophilic factor (AHF) is prepared as a multi-donor pooled component from plasma resuspended in a small volume of cryoprecipitate depleted plasma supernatant. It has the advantage over fibrinogen concentrate in that it contains constituents that impact all phases of hemostasis: von Willebrand Factor (VWF) improves primary hemostasis by enhancing platelet adhesion and aggregation at the site of vascular injury, FVIII facilitates coagulation to generate thrombin, fibrinogen forms the polymerized fibrin clot, FXIII stabilizes the fibrin clot and mitigates hyperfibrinolysis, and fibronectin improves innate immunity and stabilization of the platelet plug.^29^ In a recent ex-vivo study, Morrow, et al demonstrated that cryoprecipitate improves thrombin generation potential, increases fibrin clot stability against fibrinolytic degradation.^10^ The timing of cryoprecipitate administration is important. Because of insufficient efficacy data, cold chain requirements, and short post-thaw utility, cryoprecipitate-AHF is often given too little and too late. Indeed, at best, US massive transfusion protocols include cryoprecipitate only late in the protocol, in response to low plasma fibrinogen levels, or as an adjunct for uncontrolled bleeding or ongoing coagulopathy. Data from the PROMMTT study demonstrated that the median time from admission until cryoprecipitate administration was 2.7 hours.^30^ Logistic challenges exist in the use of conventional cryoprecipitate as it requires time and specialized equipment to thaw and once thawed, it has a shelf life limited to several hours. With these issues a lyophilized cryoprecipitate is being developed that could provide early cryo-based resuscitation en-route and in the forward environment. Clinical and logistic advantages of LPRC are rapid reconstitution, improved storage with avoidance of cold chain dependence, ability to bank products (improved surge capacity) and reduced logistic constraints. Additionally, LPRC is prepared from pathogen reduced fibrinogen complex suspended in 60-80 mL of cryoprecipitate depleted plasma supernatant containing protein C, protein S, and ADAMTS13 (<https://intercept-cryoprecipitation.com/downloads/?topic=package-insert>).

Cryoprecipitate is enriched in physiologic VWF multimers that are important in hemostasis, with over a 2-fold increase compared to plasma and a 4-fold increase compared to fibrinogen concentrate.^10^ This is in comparison to ultralarge and hyperadhesive VWF multimers released from activated endothelial cells. Importantly, Cryoprecipitate also contains a high amount of ADAMTS13 compared to plasma.^31^ We did not measure ADAMTS13 in the blood products we utilized to be able to compare to published reports but levels in mice receiving CC had almost a two-fold increase in circulating ADAMTS13 compared to FFP resuscitated mice, consistent with the difference in product levels reported by Scott et al.^31^ ADAMTS13 is a metalloenzyme that cleaves the A2 domain of intrinsically hyperadhesive ultra large VWF multimers to reduce their size and activity to levels of that of physiologic plasma VWF in the circulation.^32^  Russel et al reported that plasma levels of ADAMTS13 activity were significantly lower and VWF antigen significantly higher in severely injured pediatric patients.^12^ Similarly, MacArthur et al and Dyer et al demonstrated a reduction of ADAMTS13 in adult trauma patients.^14,16^ Additionally, the authors found that low levels of ADAMTS13 correlated with coagulopathy, transfusion requirements and injury severity, suggesting that ADAMTS 13 repletion may be beneficial.^16^  Though ADAMTS13 is not typically associated with endothelial protective effects, Kleinveld et al demonstrated in a mouse model of trauma-induced shock that recombinant ADAMTS13 reduced pulmonary endothelial permeability.^15^ Whether cryoprecipitate can adequately replete ADAMTS13 and lower pathologic VWF in injured patients has not been studied but deserves investigation and is supported by the current study. Interestingly, only CC resuscitated mice had significantly higher levels of ADAMTS13. Mice receiving LPRC and FFP had comparable (p=0.99) but levels that were lower than in mice receiving CC, but higher than LR mice. There was no significant difference in the ADAMTS13 transcription in mouse livers, suggesting the augmentation of ADAMTS13 in the CC mice was mainly due to resuscitation, though the early measurement may also be a factor. Clause, et al found in an in-vitro model that transcriptional alterations of hepatic ADAMTS13 were negligible for at least two hours after known stimulators of ADAMTS13 were added to the cells^32^. Whether changes in mRNA would occur over longer periods of time is unknown. The favorable changes seen in the ADAMTS13:VWF ratio by resuscitation with LPRC were primarily driven by lower levels of VWF, leading to a reduction in ultra-large and hyperadhesive VWF multimers released from injured endothelial cells.

There are a number of limitations of the current study. First, we only investigated one timepoint which was at three hours post resuscitation but not the immediate post-injury timepoint which may have shed more light on the dynamic changes seen after trauma. Second, neither VWF adhesive activity nor VWF multimers were measured, but VWF Ag levels have been shown by other investigators to correlate with both.^16^ Third, human ADAMTS13 may cleave mouse VWF at a different rate than in humans that could have affected our results. However, human ADAMTS13 has been shown to cleave mouse VWF in previous studies of trauma-shock, traumatic brain injury, sepsis, and thrombotic thrombocytopenic purpura (TTP)^33-36^. We also used a significantly larger volume of LR compared to product which could have led to dilutional effects on the proteins of interest we studied. Despite attempts at minimizing resuscitation volume by using a pressure rather than volume target for resuscitation and only targeting a hypotensive MAP, there was nonetheless a higher volume of LR that was required. If changes from larger volume LR were only dilutional then one would expect that both shed syndecan and VWF Ag would be lower rather than higher. Lastly, we did not directly assess if supplementing VWF and/or ADAMTS13 in the products tested were directly responsible for the improvement in endothelial cell function.

In conclusion, we have demonstrated that CC and LPRC administration were comparable to FFP in ameliorating endotheliopathy in a murine polytrauma model of hemorrhagic shock, with suggested additional benefit by LPRC. This animal data provides preliminary evidence of the safety and efficacy of LPRC and warrants further investigation for its potential application in military settings once approved for human administration.

**Supplemental Data Content includes the ARRIVE checklist.**

**References**

1. Kauvar DS, Lefering R, Wade CE. Impact of hemorrhage on trauma outcome: An overview of epidemiology, clinical presentations, and therapeutic considerations. *J Trauma - Inj Infect Crit Care*. 2006;60(6 SUPPL.):3-11.

2. Holcomb JB, Del Junco DJ, Fox EE, Wade CE, Cohen MJ, Schreiber MA, et al. The prospective, observational, multicenter, major trauma transfusion (PROMMTT) study: Comparative effectiveness of a time-varying treatment with competing risks. *JAMA Surg*. 2013;148(2):127-136.

3. Jenkins DH, Rappold JF, Badloe JF, Berséus O, Blackbourne CL, Brohi KH, et al. Trauma Hemostasis and Oxygenation Research Position Paper on Remote Damage Control Resuscitation. *Shock*. 2014;41(Supplement 1):3-12.

4. Naumann DN, Hazeldine J, Davies DJ, Bishop J, Midwinter MJ, Belli A, et al. Endotheliopathy of Trauma is an on-Scene Phenomenon, and is Associated with Multiple Organ Dysfunction Syndrome: A Prospective Observational Study. *Shock*. 2018;49(4):420-428.

5. Sperry JL, Guyette FX, Brown JB, Yazer MH, Triulzi DJ, Early-Young BJ, et al. Prehospital Plasma during Air Medical Transport in Trauma Patients at Risk for Hemorrhagic Shock. *N Engl J Med*. 2018;379(4):315-326.

6. Wu F, Kozar RA. Fibrinogen protects against barrier dysfunction through maintaining cell surface syndecan-1 in-vitro. *Shock*. 2019;51(6):740-744.

7. Wu F, Chipman A, Dong JF, Kozar RA. Fibrinogen Activates PAK1/Cofilin Signaling Pathway to Protect Endothelial Barrier Integrity. *Shock*. 2021;55(5):660-665.

8. Yu Q, Yang B, Davis JM, Ghosn J, Deng X, Doursout M-F, et al. Identification of Fibrinogen as a Key Anti-apoptotic Factor in Human Fresh Frozen Plasma for Protecting Endothelial Cells in vitro. *Shock*. 2020;53(5):646-652.

9. Caudill JSC, Nichols WL, Plumhoff EA, Schulte SL, Winters JL, Gastineau DA, et al. Comparison of coagulation factor XIII content and concentration in cryoprecipitate and fresh-frozen plasma. *Transfusion*. 2009;49(4):765-770.

10. Morrow GB, Carlier MSA, Dasgupta S, Craigen FB, Mutch NJ, Curry N. Fibrinogen replacement therapy for traumatic coagulopathy: Does the fibrinogen source matter? *Int J Mol Sci*. 2021;22(4):1-15.

11. Zeineddin A, Dong JF, Wu F, Terse P, Kozar RA. Role of Von Willebrand Factor after Injury: It May Do More Than We Think. *Shock*. 2021;55(6):717-722.

12. Russell RT, McDaniel JK, Cao W, Shroyer M, Wagener BM, Zheng XL,et al. Low Plasma ADAMTS13 Activity Is Associated with Coagulopathy, Endothelial Cell Damage and Mortality after Severe Paediatric Trauma. *Thromb Haemost*. 2018;118(4):676-687.

13. Plautz WE, Haldeman SH, Dyer MR, Sperry JL, Guyette FX, Loughran PA, et al.; A TACTIC Publication. Reduced cleavage of von willebrand factor by ADAMTS13 is associated with microangiopathic acute kidney injury following trauma. *Blood Coagul Fibrinolysis.* 2022;1;33(1):14-24.

14. Macarthur TA, Goswami J, Moon Tasson L, Tischer A, Bailey KR, Spears GM, et al. Quantification of von Willebrand factor and ADAMTS-13 after traumatic injury: A pilot study. *Trauma Surg Acute Care Open*. 2021;6(1):1-7.

15. Kleinveld DJB, Simons DDG, Dekimpe C, Deconinck SJ, Sloos PH, Maas MAW, et al.: BloodNet. Plasma and rhADAMTS13 reduce trauma-induced organ failure by restoring the ADAMTS13-VWF axis. *Blood Adv.* 2021;14;5(17):3478-3491.

16. Dyer MR, Plautz WE, Ragni MV, Alexander W, Haldeman S, Sperry JL, et al A TACTIC Publication. Traumatic injury results in prolonged circulation of ultralarge von Willebrand factor and a reduction in ADAMTS13 activity. *Transfusion.* 2020;60(6):1308-1318.

17. Peng Z, Pati S, Fontaine MJ, Hall K, Herrera AV, Kozar RA. Lack of species-specific difference in pulmonary function when using mouse versus human plasma in a mouse model of hemorrhagic shock. *J Trauma Acute Care Surg*. 2016;81(5 Suppl 2):S171-S176.

18. Lopez K, Suen A, Yang Y, Wang S, Williams B, Zhu J, et al. Hypobaria Exposure Worsens Cardiac Function and Endothelial Injury in AN Animal Model of Polytrauma: Implications for Aeromedical Evacuation. *Shock*. 2021;56(4):601-610.

19. Dyer M, Haldeman S, Gutierrez A, Kohut L, Sen Gupta A, Neal MD. Uncontrolled Hemorrhagic Shock Modeled via Liver Laceration in Mice with Real Time Hemodynamic Monitoring. *JoVE*. 2017;(123):e55554.

20. Chipman AM, Wu F, Pati S, Burdette AJ, Glaser JJ, Kozar RA. Fresh frozen plasma attenuates lung injury in a novel model of prolonged hypotensive resuscitation. *J Trauma Acute Care Surg*. 2020;89(2S Suppl 2):S118-S125.

21. Chipman AM, Wu F, Kozar RA. Fibrinogen inhibits microRNA-19b, a novel mechanism for repair of haemorrhagic shock-induced endothelial cell dysfunction. *Blood Transfus*. 2021;19(4):420-427. .

22. Hart ML, Ceonzo KA, Shaffer LA, Takahashi K, Rother RP, Reenstra WR, et al. Gastrointestinal Ischemia-Reperfusion Injury Is Lectin Complement Pathway Dependent without Involving C1q. *J Immunol*. 2005;174(10):6373-6380.

23. Pati S, Peng Z, Wataha K, Miyazawa B, Potter DR, Kozar RA. Lyophilized plasma attenuates vascular permeability, inflammation and lung injury in hemorrhagic shock. *PLoS One*. 2018;13(2).

24. Peng Z, Pati S, Potter D, Brown R, Holcomb JB, Grill R, et al. Fresh frozen plasma lessens pulmonary endothelial inflammation and hyperpermeability after hemorrhagic shock and is associated with loss of syndecan 1. Shock. 2013;40(3):195-202.

25. Zhou W, Inada M, Lee TP, Benten D, Lyubsky S, Bouhassira EE, et al. ADAMTS13 is expressed in hepatic stellate cells. *Lab Investig*. 2005;85(6):780-788.

26. Zeineddin A, Wu F, Cao S, Corash L, Pati S, Kozar RA. Immediate Use Cryoprecipitate Products Provide Lasting Organ Protection in a Rodent Model of Trauma/Hemorrhagic Shock and Prolonged Hypotensive Resuscitation. *J Trauma Acute Care Surg*. 2023; Accepted for publication.

27. Wu F, Chipman A, Pati S, Miyasawa B, Corash L, Kozar RA. Resuscitative strategies to modulate the endotheliopathy of trauma: From cell to patient. *Shock*. 2020;53(5):575-584.

28. Barry M, Trivedi A, Miyazawa BY, Vivona LR, Khakoo M, Zhang H, et al. Cryoprecipitate attenuates the endotheliopathy of trauma in mice subjected to hemorrhagic shock and trauma. *J Trauma Acute Care Surg*. 2021;90(6):1022-1031.

29. Cushing MM, Haas T, Karkouti K, Callum J. Which is the preferred blood product for fibrinogen replacement in the bleeding patient with acquired hypofibrinogenemia—cryoprecipitate or fibrinogen concentrate? *Transfusion*. 2020;60(S3):S17-S23.

30. Holcomb JB, Fox EE, Zhang X, White N, Wade CE, Cotton BA, et al. Cryoprecipitate Use in the Prospective Observational Multicenter Major Trauma Transfusion study (PROMMTT) on behalf of the PROMMTT Study Group. *J Trauma Acute Care Surg*. 2013;75(1):31-39.

31. Scott EA, Puca KE, Pietz BC, DuChateau BK, Friedman KD. Comparison and stability of ADAMTS13 activity in therapeutic plasma products. *Transfusion*. 2007;47(1):120-125.

31. Furlan M, Robles R, Lämmle B. Partial purification and characterization of a protease from human plasma cleaving von Willebrand factor to fragments produced by in vivo proteolysis. *Blood*. 1996;87(10):4223-4234.

32. Claus RA, Bockmeyer CL, Kentouche K, Sieber MW, Oberle V, Kaufmann R, et al. Transcriptional regulation of ADAMTS13. *Thromb Haemost*. 2005;94(1):41-5.

33. Kleinveld DJB, Simons DDG, Dekimpe C, Deconinck SJ, Sloos PH, Maas MAW, et al.; BloodNet. Plasma and rhADAMTS13 reduce trauma-induced organ failure by restoring the ADAMTS13-VWF axis. *Blood Adv*. 2021;14;5(17):3478-3491.

34. Wu Y, Liu W, Zhou Y, Hilton T, Zhao Z, Liu W, et al. Von Willebrand factor enhances microvesicle-induced vascular leakage and coagulopathy in mice with traumatic brain injury. *Blood*. 2018;132(10):1075-1084.

35. Lerolle N, Dunois-Lardé C, Badirou I, Motto DG, Hill G, Bruneval P, et al. von Willebrand factor is a major determinant of ADAMTS-13 decrease during mouse sepsis induced by cecum ligation and puncture. *J Thromb Haemost.* 2009;7(5):843-50.

36. Schiviz A, Wuersch K, Piskernik C, Dietrich B, Hoellriegl W, Rottensteiner H, et al. A new mouse model mimicking thrombotic thrombocytopenic purpura: correction of symptoms by recombinant human ADAMTS13. *Blood*. 2012;119(25):6128-35.

**Figure Legends:**

**Figure 1. Mean arterial pressure after polytrauma and hypotensive resuscitation is improved by resuscitation with cryoprecipitate products.** Mice underwent polytrauma with muscle crush and tibia fracture followed by laparotomy and liver injury with 60 minutes of uncontrolled hemorrhagic shock then resuscitation to a MAP of 55-60 mm Hg for 3 hours with Lactated Ringers (LR), Fresh Frozen Plasma (FFP), Conventional Pathogen-Reduced Cryoprecipitate (CC), and Lyophilized Pathogen-Reduced Cryoprecipitate (LPRC) and compared to shams. Blood pressure is shown at baseline, abdominal closure after liver laceration (BP @ abd closure), 30 minutes after reaching shock state (BP @ 30), 60 minutes after reaching shock state which was also just prior to the start of resuscitation (pre-res BP @60), then at 60, 120, and 180 minutes after resuscitation (post-res BP). Results were analyzed by ANOVA with Tukey post hoc with multiple comparisons corrections; n=9-11/group.

**Figure 2**. **Lung injury and permeability reduced by cryoprecipitate products**. Mice underwent polytrauma with muscle crush and tibia fracture followed by laparotomy and liver injury with 60 minutes of uncontrolled hemorrhagic shock then resuscitation to a MAP of 55-60 mm Hg for 3 hours with Lactated Ringers (LR), Fresh Frozen Plasma (FFP), Conventional Pathogen-Reduced Cryoprecipitate (CC), and Lyophilized Pathogen-Reduced Cryoprecipitate (LPRC) and compared to shams. Lung tissue and bronchoalveolar lavage (BAL) collected at the time of euthanasia. **Upper panel)** Lung Histopathologic Injury. Shown are representative images and the corresponding lung injury scores and **Lower panel**.) Lung bronchial alveolar lavage (BAL) protein as an indicator of permeability. Data is reported as mean ±SD, n=5-8 /group and analyzed by one-way ANOVA with Bonferroni post hoc; **p<0.05 vs sham; * p<0.05 vs LR.

**Figure 3. Lung syndecan-1 immunostaining enhanced by cryoprecipitate products.** Mice underwent polytrauma with muscle crush and tibia fracture followed by laparotomy and liver injury with 60 minutes of uncontrolled hemorrhagic shock then resuscitation to a MAP of 55-60 mm Hg for 3 hours with Lactated Ringers (LR), Fresh Frozen Plasma (FFP), Conventional Pathogen-Reduced Cryoprecipitate (CC), and Lyophilized Pathogen-Reduced Cryoprecipitate (LPRC) and compared to shams. Lung tissue was stained with anti-mouse syndecan-1 antibody and Alexa Fluor 488 goat anti-mouse IgG and imaged with a fluorescent microscope. Shown are representative images and the corresponding relative fluorescent units (RFUs). Data is reported as mean ±SD, n= 4/group with a minimum of three images per animal and analyzed by one-way ANOVA with Bonferroni post hoc. **p<0.05 vs sham; * p<0.05 vs LR.

**Figure 4.** **Systemic syndecan shedding was lessened by cryoprecipitate products**. Mice underwent polytrauma with muscle crush and tibia fracture followed by laparotomy and liver injury with 60 minutes of uncontrolled hemorrhagic shock then resuscitation to a MAP of 55-60 mm Hg for 3 hours with Lactated Ringers (LR), Fresh Frozen Plasma (FFP), Conventional Pathogen-Reduced Cryoprecipitate (CC), and Lyophilized Pathogen-Reduced Cryoprecipitate (LPRC) and compared to shams. Blood was obtained at the time of euthanasia for measurement of syndecan-1 by ELISA. Data is reported as mean ±SD, n= 5-8/group and analyzed by one-way ANOVA with Bonferroni post hoc. **p<0.05 vs sham; * p<0.05 vs LR.

**Figure 5. Ratio of ADAMTS13:VWF is improved by cryoprecipitate products.** Mice underwent polytrauma with muscle crush and tibia fracture followed by laparotomy and liver injury with 60 minutes of uncontrolled hemorrhagic shock then resuscitation to a MAP of 55-60 mm Hg for 3 hours with Lactated Ringers (LR), Fresh Frozen Plasma (FFP), Conventional Pathogen-Reduced Cryoprecipitate (CC), and Lyophilized Pathogen-Reduced Cryoprecipitate (LPRC) and compared to shams. Blood was obtained at the time of euthanasia for measurement of VWF and ADAMTS13 by ELISA. Shown is the ratio of ADAMTS13: VWF Ag. n=4-8/group. **p<0.05 vs sham; * p<0.05 vs LR.

**Table 1. Physiologic parameters across experimental groups**

| **Group** | **N** | **Mortality (%)** | **Volume of hemoperitoneum (µl)** | **Blood removed to achieve shock state (µl)** | **Volume resuscitation in µl (ml/kg)** |
| --- | --- | --- | --- | --- | --- |
| Sham | 17 | 0 | N/A | N/A | N/A |
| LR | 15 | 5 (33%) | 676±211 | 262±94 | 1270±340 (43±10) |
| FFP | 14 | 3 (14%) | 634±180 | 302±96 | 364±106 (11±3) |
| CPRC | 15 | 5 (33%) | 592±254 | 358±127 | 337±108 (11±4) |
| LPRC | 13 | 4 (31%) | 729±222 | 273±174 | 481±156 (16±5) |
| **p value** |  | NS | NS | NS | <0.01 |

LR=Lactated Ringers, FFP= fresh frozen plasma; CPRC=conventional pathogen-reduced cryoprecipitate; LPRC=lyophilized pathogen-reduced cryoprecipitate
