# Supplementary material for: Early lyophilized cryoprecipitate enhances the ADAMTS13/VWF ratio to reduce systemic endotheliopathy and lessen lung injury in a mouse multiple-trauma hemorrhage model
Source: J Trauma Acute Care Surg. 2023 May 22;95(2):S137–43. doi: 10.1097/TA.0000000000004065 (PMC10389395; doi:10.1097/TA.0000000000004065)
Supplement: Supplementary file 2 [file jt-95-s137-s002.pptx]

## Slide 1
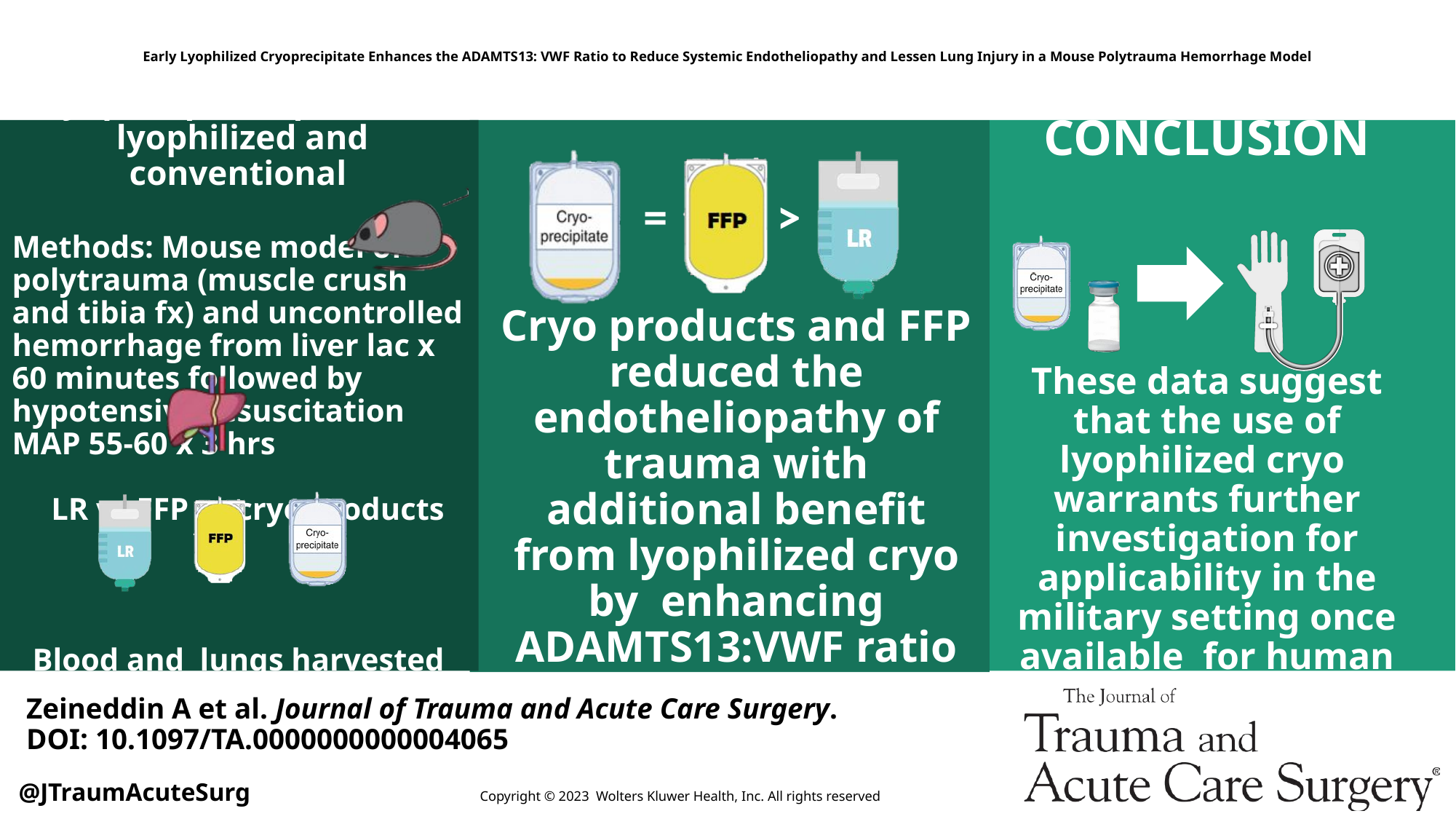

# Early Lyophilized Cryoprecipitate Enhances the ADAMTS13: VWF Ratio to Reduce Systemic Endotheliopathy and Lessen Lung Injury in a Mouse Polytrauma Hemorrhage Model
RESULTS
Cryo products and FFP reduced the endotheliopathy of trauma with additional benefit from lyophilized cryo by enhancing ADAMTS13:VWF ratio
= >
Cryoprecipitate products: lyophilized and conventional
Methods: Mouse model of polytrauma (muscle crush and tibia fx) and uncontrolled hemorrhage from liver lac x 60 minutes followed by hypotensive resuscitation MAP 55-60 x 3 hrs
 LR vs FFP vs cryo products
Blood and lungs harvested
after 3 hours
Conclusion
These data suggest that the use of lyophilized cryo warrants further investigation for applicability in the military setting once available for human use
Zeineddin A et al. Journal of Trauma and Acute Care Surgery.
DOI: 10.1097/TA.0000000000004065
 @JTraumAcuteSurg Copyright © 2023 Wolters Kluwer Health, Inc. All rights reserved
