# Supplementary material for: Early lyophilized cryoprecipitate enhances the ADAMTS13/VWF ratio to reduce systemic endotheliopathy and lessen lung injury in a mouse multiple-trauma hemorrhage model
Source: J Trauma Acute Care Surg. 2023 May 22;95(2):S137–43. doi: 10.1097/TA.0000000000004065 (PMC10389395; doi:10.1097/TA.0000000000004065)
Supplement: Supplementary file 4 [file jt-95-s137-s004.doc]

Dear Author,

Please review the fees to produce your figures in color in *The Journal of Trauma*. The price for the first color figure is $750. The charge for each additional color figure is $150.

If this charge meets with your approval, please identify which figures/pieces should be printed in color, sign below, and email a copy of this letter to my attention. If your institution or affiliation will cover the cost, please have your purchasing agent sign below. You will be invoiced for all article charges (color separations, reprints, etc.) after publication.

If you do not wish your figures to run in color, please indicate, “Decline color” below and return a copy of this letter to me by email. The figures will then run in black and white.

Please return this letter at your soonest possible convenience. Prompt return of the signed form, even if you choose to decline color, will avoid delays in publication.

Thank you.

Meagan Wilson

Production Editor

Lippincott Williams & Wilkins

[Meagan.Wilson@wolterskluwer.com](mailto:Megan.Hein@wolterskluwer.com)

□X AAST, EAST, WTA and PTS members (receive complimentary color)

Member number _AAST **15671 (never seen membership number; also member of WTA and EAST**__________

Figures to appear in color: __Figure 2 and 3________________________

Total cost for color figures: _____zero __** if there are any issues then would decline color____________

Article Number: _____JT-D-23-00098R1 _____________________

Billing information: not applicable

__________________________________

Author’s Name or Institution/Affiliation

______________________________________________________________________________________

Address

_____________________________

Printed Name

_____________________________________________

Signature Date

🞏 **Decline Color**
